# PSORTdb: expanding the bacteria and archaea protein subcellular localization database to better reflect diversity in cell envelope structures

**DOI:** 10.1093/nar/gkv1271

**Published:** 2015-11-23

**Authors:** Michael A. Peabody, Matthew R. Laird, Caitlyn Vlasschaert, Raymond Lo, Fiona S.L. Brinkman

**Affiliations:** 1 Department of Molecular Biology and Biochemistry, Simon Fraser University, Burnaby, British Columbia, V5A 1S6, Canada; 2 Department of Biochemistry, Microbiology and Immunology, University of Ottawa, Ottawa, Ontario, K1N 6N5, Canada

## Abstract

Protein subcellular localization (SCL) is important for understanding protein function, genome annotation, and has practical applications such as identification of potential vaccine components or diagnostic/drug targets. PSORTdb (http://db.psort.org) comprises manually curated SCLs for proteins which have been experimentally verified (ePSORTdb), as well as pre-computed SCL predictions for deduced proteomes from bacterial and archaeal complete genomes available from NCBI (cPSORTdb). We now report PSORTdb 3.0. It features improvements increasing user-friendliness, and further expands both ePSORTdb and cPSORTdb with a focus on improving protein SCL data in cases where it is most difficult—proteins associated with non-classical Gram-positive/Gram-negative/Gram-variable cell envelopes. ePSORTdb data curation was expanded, including adding in additional cell envelope localizations, and incorporating markers for cPSORTdb to automatically computationally identify if new genomes to be analysed fall into certain atypical cell envelope categories (i.e. Deinococcus-Thermus, Thermotogae, Corynebacteriales/Corynebacterineae, including *Mycobacteria*). The number of predicted proteins in cPSORTdb has increased from 3 700 000 when PSORTdb 2.0 was released to over 13 000 000 currently. PSORTdb 3.0 will be of wider use to researchers studying a greater diversity of monoderm or diderm microbes, including medically, agriculturally and industrially important species that have non-classical outer membranes or other cell envelope features.

## INTRODUCTION

Identification of protein subcellular localization (SCL) aids in understanding the function of proteins, determining its potential interaction partners and identifying cell surface-exposed components. Bacterial and archaeal proteins can exist freely in cellular spaces such as the cytoplasm or periplasmic space, anchored in cytoplasmic or outer membranes, excreted into the extracellular space, or even injected directly into eukaryotic/host cells. The determination or prediction of SCL to any outer membrane, S-layer (surface layer), or extracellular environment (secreted), is of particular interest. These proteins may be more accessible to the immune system, so be of interest as potential vaccine components, and their accessibility also makes them more attractive as potential drug targets and for use in microbial diagnostics with medical or non-medical applications (1–4).

Determination of the SCL of proteins through low-throughput laboratory experiments is accurate, providing high-quality localization information, but is laborious and expensive. High-throughput laboratory methods, involving subcellular fractionation and proteomics, are rapid and relatively cost-effective. However, they are notably less accurate, in particular due to cross-contamination of cellular sub-fractions (5,6). Computational/*in silico* SCL prediction methods require only the genome (or gene) sequence and high-precision computational SCL predictors, such as PSORTb (7–9), have been shown to exceed the accuracy of common high-throughput laboratory approaches (6).

**Figure 1.**
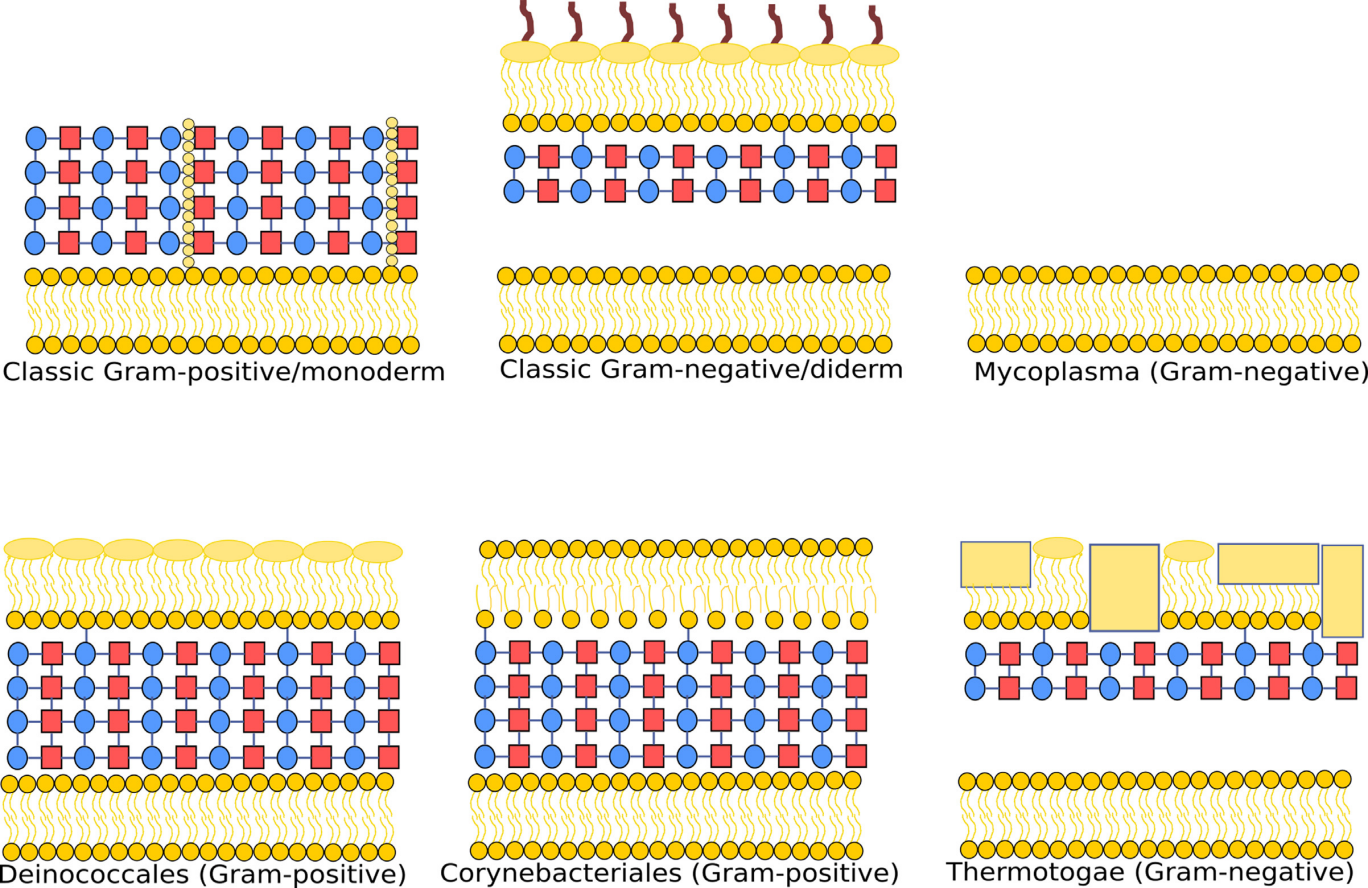
Schematic illustrating the diversity of arrangements for bacterial cell envelopes, with selected examples. Classic Gram-positive bacteria are monoderms (contain one cell membrane in their cell envelope) with a thick peptidoglycan layer, and classic Gram-negative bacteria are diderms (enveloped by two membranes) with a thin peptidoglycan layer between the inner and outer membranes. Examples of bacteria with other cell envelope structures include *Mycoplasma* (monoderms without a peptidoglycan layer), Deinococcales (diderms with a thick peptidoglycan layer that lacks lipopolysaccharide), Corynebacteriales (diderms with a thick peptidoglycan layer and unique outer membrane containing mycolic acids also known as a mycomembrane) and Thermotogae (diderms with a unique outer membrane, called a toga, which is very rich in proteins). These schematic diagrams do not show certain components such as S-layers. Red squares represent *N*-acetylglucosamine, blue circles represent *N*-acetylmuramic acid, beige squares represent proteins and lipopolysaccharides are shown in brown.

Although there are a variety of cellular envelope structures, most SCL predictors have focused on predictions for just the two most common types of cell envelope arrangements—the classic Gram-positive monoderms (one cell membrane) and Gram-negative diderms (enveloped by two cell membranes; Figure 1) (4). Classic Gram-positive bacteria primarily comprise the cytoplasm, the cytoplasmic membrane and a cell wall surrounding the cytoplasmic membrane containing a thick layer of peptidoglycan. Many of the most well-studied Archaea contain these same basic components as classic Gram-positive bacteria. Classic Gram-negative bacteria, however, comprise the cytosol, cytoplasmic membrane, an additional outer membrane and between the two membranes a periplasmic space that contains a thin cell wall composed of peptidoglycan. Although many of the well-studied bacteria conform to the classic Gram-positive monoderm and Gram-negative diderm cell types, there are notable exceptions (10). Corynebacteriales, which includes notable pathogens *Mycobacterium tuberculosis* and *Mycobacterium leprae* (11), have a completely different type of outer membrane, composed of mycolic acids that stains Gram-negative or Gram-variable, even though they contain a thick peptidoglycan layer (12). Some ‘atypical Gram-positives’ stain Gram-positive due to a thick peptidoglycan layer, but also have an outer membrane, such as Deinococcales which include *Deinococcus spp*. (13). There are also atypical Gram-negatives that stain Gram-negative due to the reduced/lack of peptidoglycan cell wall, but also have no outer membrane, such as Mollicutes which include pathogens within the *Mycoplasma spp*. (14). There are additionally atypical Gram-negative bacteria which have a non-classical Gram-negative outer membrane. For example the Thermotogae have a unique outer membrane, also known as a toga, which is very different from the classic Gram-negative outer membrane. This toga is likely responsible for their hyperthermophilicity (10), and is very rich in proteins (15). A schematic overview of these different types of cell envelopes is shown in Figure 1.

There exist several databases containing prokaryotic SCL information. Some of these databases contain general protein annotations which include SCL, such as UniProt (16). Several others, such as CoBaltDB (17), incorporate predictions from multiple SCL tools. There also exist databases that are targeted towards specific interests. For example, DBMLoc (18) is a database specific for proteins with multiple SCLs. Other SCL databases are specific for certain types of bacteria, such as the LocateP database (19) specific to Gram-positive bacteria, or ClubSub-P which contains predictions only for Gram-negative bacteria and Archaea (20). Although there are many databases containing SCL information, there is a need to improve support for prokaryotes with diverse cell envelope structures. PSORTdb (21), first released in 2005, is a bacterial and archaeal SCL database comprising two components: ePSORTdb, which contains experimental determined, manually curated protein SCLs, and cPSORTdb, which contains computationally predicted protein SCLs derived from the SCL predictor PSORTb (7–9). The original version included only predictions for Gram-positive and Gram-negative bacteria, but PSORTdb 2.0 published in 2011 (22) expanded to include all predictions made by a new version of PSORTb that included Archaea, and also was set up to have automatic updates. This database has continued to be updated over the years; however, the coverage of these bacteria with diverse cell envelopes was relatively limited, for example, initially containing only 44 replicons belonging to bacteria with atypical cell envelope structures in cPSORTdb. Organisms such as medically important *Mycobacteria* did not have certain key localizations predicted, such as the mycobacterial outer membrane proteins. There was a high need to improve the database to better handle the diversity of SCLs that may be present in the Bacteria and Archaea.

Here we describe PSORTdb 3.0 (http://db.psort.org/), an expanded database that better reflects the diversity in cell envelope structures, and ensures certain proteins of high medical interest are predicted appropriately. It builds upon PSORTdb 2.0 by including some additional user friendly features, additional manually curated annotations of proteins from bacteria with atypical cellular envelopes in ePSORTdb, and updated computationally predicted SCLs in cPSORTdb, utilizing the new ePSORTdb protein annotations and expanded SCL prediction categories. Furthermore, we have improved our automated update system, adding in the computational predictor required to automatically detect bacteria with certain atypical cell envelope structures. This database will be of particular interest to researchers studying microbes outside those with the classical Gram-negative diderm and Gram-positive monoderm cell envelope structures, including medically relevant species such as *Mycobacterium tuberculosis* and *Mycoplasma pneumoniae* (23,24), agriculturally relevant species such as *Spiroplasma citri* (25) and industrially relevant species such as *Thermotoga maritima* (26).

### User-friendly database features, and expanded subcellular localizations to better reflect bacteria with non-classical bacterial and archaeal subcellular localization

To better reflect bacterial diversity we have incorporated additional subcellular localization categories. In particular the ‘toga’ subcategorization/secondary localization was to highlight the notably unique structure which is a particularly protein rich envelope layer. We also specify when proteins are predicted to be in the S-layer, which is an additional subcategorization. However, note that we have continued to maintain the main localization sites used in previous versions of this database: cytoplasmic, cytoplasmic membrane, periplasm, cell wall, outer membrane and extracellular. This is critical to ensure analyses of SCL maintain stability across database versions and enable appropriate comparisons across both PSORTdb versions and with other SCL databases. Any expanded subcellular localizations are classified under a subcategory system, to provide finer resolution predictions and highlight notable differences. For example, proteins in the Thermotogae outer membrane type structure with the subcategory name ‘toga’ help the PSORTdb database user appreciate that the Thermotogae does not have a classical outer membrane, but rather has a specialized, very unique, ‘toga’ one. Note though that some organisms such as Deinococcales, have outer membranes that are simply referred to as such, even though they are not classical Gram-negatives, as that is what they are referred to as. In addition to these notable subcategory SCLs added, more user-friendly features have been incorporated into the database. We have made changes to the site to help clarify certain points such as providing educational material on the different types of cell envelope structures and localizations, separating out the cPSORTdb and ePSORTdb search pages for a more customized search environment for each, and clarifying ways to search (i.e. cPSORTdb searches can be made by just genome, or more sophisticated searches on various fields, but one should be aware that multiple genomes can have the same strain name now in NCBI, and so an advanced search with a strain name can return results from multiple proteomes. In cases where one wants to identify, for example, outer membrane proteins associated with a particular strain, a genome-specific search, which can then be limited to different organisms, may be preferred). Additionally, there is now greatly increased ease of local installation, should one want to locally run genomes not available in our automated updates. We created a Docker installation of PSORTb which can be found at https://github.com/brinkmanlab/psortb-docker (previously, running PSORTb locally required multiple dependencies and was distinctly not a user-friendly installation). We also increased the user-friendliness of certain Ajax-based searches of the database, either by protein or by genome, to better allow more complex queries.

### Expanded ePSORTdb database of proteins with experimentally determined SCL, with a focus on key proteins found in bacteria with atypical cell envelope structures

We have expanded the curated ePSORTdb database with additional entries for bacteria with atypical cell envelope structures, which came from manual literature search. In addition to more classical organisms, there are now 143 entries covering key proteins known in Gram-negative bacteria without an outer membrane, 55 entries reflecting targeted proteins found in Gram-positive bacteria with an outer membrane and 56 entries for Thermotogae which have an additional atypical outer membrane. These entries will be of interest to researchers studying these bacteria with diverse cellular envelopes, as well as for bioinformaticists interested in training data for developing SCL predictors.

### Incorporation of a more flexible computational predictor for identifying atypical cell envelope structure for cPSORTdb update computations

In PSORTdb version 2, we introduced a computational outer membrane detection procedure relying on Omp85, the only essential outer membrane protein found in all classic Gram-negative bacteria (22,27). This greatly enabled automatic updating of SCL predictions from deduced proteomes of complete genomes, by enabling automated prediction of which microbial genomes should be run as a ‘Gram-positive’ and which should be run with the ‘Gram-negative’ designation for PSORTb. However, this procedure did not detect all outer membranes such as the unusual outer membrane of Corynebacteriales (including medically relevant *Mycobacteria*), nor did it distinguish between other outer membranes, such as the toga of Thermotogae. Thus, we incorporated markers for automated detection of such atypical outer membrane/cell envelope types, so the deduced proteomes from microbial genomes may be correctly assigned the right PSORTb modules and thus correct SCL predictions made.

Corynebacteriales are found in the traditionally Gram-positive phylum Actinobacteria, and contain a non-traditional waxy outer membrane. This outer membrane resists Gram staining or is Gram-variable / acid-fast, even though they contain a thick peptidoglycan cell wall. This very different outer membrane does not contain Omp85, which is not surprising, as Corynebacteriales is believed to be the result of convergent evolution of an outer membrane (28). This is believed not only because the Corynebacteriales are within the phylum Actinobacteria where the rest of the known microbes have classic Gram-positive cell envelopes, but also because the composition and biogenesis of the outer membrane is completely different from traditional classic Gram-negative outer membranes (28). We have identified a signature marker for Corynebacteriales, a cutinase (29), which was found to be conserved uniquely within Corynebacteriales. This marker has been incorporated into PSORTdb, which acts alongside the Omp85 outer membrane detector. First, the Omp85 detector is run to identify if the organism contains a classical outer membrane, and if they do not, the cutinase detector is run to identify if the organism contains a Corynebacteriales mycolic acid containing outer membrane. This predictor may be useful for rapidly identifying the membrane structure of newly sequenced bacterial genomes.

Although we attempted to also find markers for automated detection of other atypical cell envelope types such as those of the Thermotogae and Deinococcales, we were unable to identify markers that we were highly confident covered these groups of bacteria at this time. Therefore, we decided to continue to rely on the NCBI taxonomy which is in most cases a good indicator of cell envelope structure, though it does not contain any such data to identify microbial cell envelope types. Previously, the phylum of a genome was used as the indicator of cell envelope structure. However, not all members of a phylum necessarily share the same cell envelope structure. For example, the Deinococcales stain Gram-positive but have an outer membrane, while the Thermales within the same phylum Deinococcus-Thermus stains Gram-negative. We have updated our method that classifies cell envelope structure based on taxonomy, so that it is more flexible and can now classify based on any level of the taxonomy, allowing different cell envelope structure classifications within the same phylum. We aim to continue to expand this database feature, to accommodate additional bacterial and archaea with atypical cell envelope structures as they are discovered, including the use of markers for rapid automatic identification of specific types of atypical cell envelope structures.

### Expanded cPSORTdb database for all bacteria and archaea that have complete genome sequences, incorporating expanded localization subcategories

cPSORTdb has been updated as more bacterial and archaeal genome sequences have become available through NCBI's microbial genome database and our associated MicrobeDB (30). As part of this process, MicrobeDB needed to be extensively updated to reflect changes in NCBI database structure which it would access. There are now SCL predictions for the deduced proteomes from over 1917 Gram-positive replicons, 5042 Gram-negative replicons (including 31 Thermotogae), 245 archaeal replicons, 123 replicons that belong to Gram-negative bacteria without an outer membrane and 287 replicons that belong to Gram-positive bacteria with an outer membrane. In total, there are over 13 000 000 proteins with predicted SCL, with the total number of proteins predicted for each SCL summarized in Table 1. By examining the number of predictions (everything but unknown) relative to the total number of proteins, we can identify the coverage of proteins, or proportion of proteins that get a predicted SCL by cPSORTdb. For Gram-positive organisms, we have 2 956 985 proteins with a predicted SCL out of 3 658 804 total proteins, for an average coverage of 80.8%. There is 71.7% coverage for Gram-negative bacteria (6 181 044/ 8 619 991), 86.8% coverage for Archaea (327 046/376 790), 65.9% coverage for Gram-negative bacteria without an outer membrane (60 948/92 512) and 68.4% coverage for Gram-positive bacteria with an outer membrane (510 605/746 232). The lower overall average coverage for bacteria with atypical cell envelopes indicates that, even with SCL entries in ePSORTdb which were incorporated into the predictor for cPSORTdb, there is still a need for additional work to be done to improve predictions for these less well studied bacteria. This may be done by gathering more experimental data, incorporating additional proteins of known SCL into the training sets of these predictors, or by the design of computational predictors (or modules of predictors) that specialize in predicting the SCL of proteins from bacteria with atypical cell envelope structures.

**Table 1.** Total number of proteins for each computationally predicted SCL site currently in the cPSORTdb data set, grouped by type of microbe

SCL site	Gram-positive	Gram-negative^a^	Archaea	Advanced-^b^	Advanced+^c^
Cytoplasmic	1 852 434	3 792 447	249 648	37 926	333 168
Cytoplasmic Membrane	1 004 086	1 882 975	72 249	21 326	153 925
Cell wall	39 244	-	1702	-	-
Extracellular	61 221	100 158	3447	1696	9420
Outer Membrane	-	175 500	-	-	1950
Periplasmic	-	229 964	-	-	12 142
Unknown	701 819	2 438 947	49 744	31 564	235 627

^a^Classic Gram-negative, plus those with atypical outer membranes, such as the toga in Thermotogae, which are denoted by additional subcategorization as mentioned in the text.

^b^Gram-negative without an outer membrane.

^c^Gram-positive or Gram-variable, with an outer membrane.

## CONCLUSION

Genome sequencing has become substantially cheaper and quicker over the previous few years, with the number of sequences deposited in publically available databases increasing exponentially. Thus, the use of computational methods for annotation of genomes, such as predictors of SCL, has become increasingly important. This is even more pressing as new computational approaches for development of vaccines and diagnostics depend on accurate SCL prediction. A new version of PSORTdb has been developed which incorporates a more flexible computational predictor for identifying a wider variety of atypical cell envelope structures, an expanded data set of experimentally verified SCLs found in bacteria with atypical cell envelope structure, some improved database interface features, and an up-to-date data set of computationally predicted SCLs with expanded localization subcategories. This update, which expands the capabilities of PSORTdb to predict the protein SCLs from bacteria with more diverse cell envelope structures, will be of wide interest to researchers studying a diversity of microbes, including notable medically important microbes belonging to the Mycobacteriaceae, and organisms of industrial interest within the Thermotogae. As more diverse cell envelope structures are identified, including additional outer membranes anticipated that likely have evolved by convergent evolution, the database is well structured now to balance both predicting primary SCLs, as well as handling more diverse or specific subcategory localizations in keeping with more diverse cell structures.
